# Resuscitation with low volume hydroxyethylstarch 130 kDa/0.4 is not associated with acute kidney injury

**DOI:** 10.1186/cc8920

**Published:** 2010-03-18

**Authors:** Nicolas Boussekey, Raphaël Darmon, Joachim Langlois, Serge Alfandari, Patrick Devos, Agnes Meybeck, Arnaud Chiche, Hugues Georges, Olivier Leroy

**Affiliations:** 1 Intensive care and infectious disease unit, Tourcoing hospital, 135, rue du Président Coty Tourcoing BP 619, 59208 France; 2 CERIM, University of Lille, Place de Verdun, Lille 59000 France

## Abstract

**Introduction:**

Acute kidney injury (AKI) in the ICU is associated with poorer prognosis. Hydroxyethylstarch (HES) solutions are fluid resuscitation colloids frequently used in the ICU with controversial nephrotoxic adverse effects. Our study objective was to evaluate HES impact on renal function and organ failures.

**Methods:**

This observational retrospective study included 363 patients hospitalized for more than 72 hours in our ICU. A hundred and sixty eight patients received HES during their stay and 195 did not. We recorded patients' baseline characteristics on admission and type and volume of fluid resuscitation during the first 3 weeks of ICU stay. We also noted the evolution of urine output, the risk of renal dysfunction, injury to the kidney, failure of kidney function, loss of kidney function and end-stage kidney disease (RIFLE) classification and sepsis related organ failure assessment (SOFA) score over 3 weeks.

**Results:**

Patients in the HES group were more severely ill on admission but AKI incidence was similar, as well as ICU mortality. The evolution of urine output (*P *= 0.74), RIFLE classification (*P *= 0.44) and SOFA score (*P *= 0.23) was not different. However, HES volumes administered were low (763+/-593 ml during the first 48 hours).

**Conclusions:**

Volume expansion with low volume HES 130 kDa/0.4 was not associated with AKI.

## Introduction

Hydroxyethylstarches (HES) are resuscitation solutes largely employed in intensive care units (ICU) [1]. However, their potential nephrotoxic effect is controversial [2-18] and acute kidney injury (AKI) in the ICU is associated with a 60% mortality rate [19]. The 2001 prospective randomized study by Schortgen and colleagues [5] showed that plasma volume expansion with HES was an independent risk factor for AKI compared with gelatins. More recently, the volume substitution and insulin therapy in severe sepsis (VISEP) study [6] compared ringer's lactate with HES 200 kDa/0.5 for fluid resuscitation in patients with severe sepsis or septic shock. HES use was associated with renal failure and increased need for renal replacement therapy (RRT). Renal failure was directly related to the volume of HES administered with a dose-effect relation. Coupled with the results of another recent study [7], some experts addressed the question of the continuing usefulness of HES use in the ICU [9]. However, published studies compared HES with different molecular weights, degrees of substitution and diluents and used variable definitions of kidney failure. Therefore, we decided to conduct a practice survey including all the patients hospitalized in our ICU during a two-year period to evaluate if plasma volume expansion with a 'modern' HES 130 kDa/0.4 had an impact on kidney function according to the validated RIFLE (Risk of renal dysfunction, Injury to the kidney, Failure of kidney function, Loss of kidney function and End-stage kidney disease) classification.

## Materials and methods

### Inclusion criteria and study goal

We included all the patients hospitalized for the first time for more than 72 hours in the ICU of Tourcoing Hospital, France, from July 2006 to July 2008. As it was a retrospective study, in accordance with French law, neither approval of the ethics committee nor informed consent was required.

We evaluated the impact of volume expansion with HES 130 kDa/0.4 on the evolution of renal function and other organ failures during the first three weeks of patients' stay in our unit.

### Data collection and definitions

#### On admission

For all the patients, the following characteristics were collected on ICU admission: age, gender, underlying clinical conditions, severity of illness and vital sign abnormalities. In particular, we examined if patients presented with chronic kidney disease (defined by a creatinine clearance <60 ml/min), and had more than one cardiovascular underlying disease (myocardial infarction, stroke, lower limb arteritis). We checked if patients had systemic inflammatory reaction syndrome, sepsis or septic shock, or shock from other origin. Shock was defined as a sustained (one hour or more) decrease in the systolic blood pressure of at least 40 mmHg from baseline or a resultant systolic blood pressure less than 90 mmHg after adequate fluid replacement and in the absence of any antihypertensive drug [20]. Severity of illness was assessed by the Sepsis related Organ Failure Assessment (SOFA) score [21] and the Simplified Acute Physiology Score (SAPS) II [22]. Potential nephrotoxic factors were recorded, including usual treatment by diuretics, angiotensin-converting enzyme (ACE) inhibitors, angiotensin-II receptor blockers (ARBs) or non-steroidal anti-inflammatory drugs (NSAIDs).

### Evolution

Volume expansion was quantified every 48 hours until ICU discharge or for a maximum of three weeks. We collected data on HES 130 kDa/0.4, crystalloids, 4% albumin and packed red blood cells (dextrans and gelatins are not used in our unit and our starch is a non-balanced solution). Plasma volume expansion did not follow a protocol. The evolution of the SOFA score, urine output and RIFLE [23] classification was recorded every 48 hours during three weeks.

To evaluate the evolution of renal failure, we analyzed two groups of patients: those with normal kidney function and those with RIFLE class 'risk' on admission. Patients were considered to have AKI when the RIFLE class increased to 'injury' or 'failure' during the three-week follow up.

Development of a nosocomial infection or a new shock between day 3 and day 21 was recorded. Potential AKI risk factors were also noted: use of nephrotoxic antibiotics (aminoglycosides, glycopeptides), inappropriate administration (aminoglycosides injection whereas plasma measurement was superior to 2 μg/ml, vancomycinemia superior to 30 μg/ml), exposure to radio-contrast agents, rhabdomyolysis defined by creatine phospho kinase (CPK) measurement superior to five times the normal, urinary obstruction, or treatment with amphotericin B or nephrotoxic chemotherapy (cisplatin or cyclosporine). Treatment with aminoglycosides did not exceed three days. Prognostic values were recorded: number of days under mechanical ventilation, vasopressors and RRT on day 28. Mortality was recorded in the ICU only.

### Statistical analysis

Comparisons between groups were performed using chi-squared test or Fisher's exact test for categorical parameters. Continuous variables were analysed using Wilcoxon's test. Differences between groups were considered to be significant for variables yielding a *P *value less than 0.05. The evolutions of urine output, SOFA score and RIFLE class were analyzed with a linear mixed model. All analyses were performed using the SAS Software (SAS Institute, Version 8.2 ed. Cary, North Carolina, USA).

## Results

Between July 2006 and July 2008, 363 patients were hospitalized for more than 72 hours in our ICU. One hundred and sixty-eight patients (46% of the population) received HES during the three-week follow up. Volume expansion with crystalloids and albumin was similar in the two groups (Table 1). We only noticed more packed red blood cells transfusions at 48 hours in the HES group. Half the HES volume (763 ± 595 ml) was administered in the first 48 hours of ICU stay. Baseline characteristics differed in patients who did and did not receive HES. Particularly, patients who received HES had more frequent surgical admissions (23.1% vs. 11.7%, *P *< 0.01), higher severity scores (SAPS II: 53.5 ± 17.1 vs. 46.6 ± 16.8, *P *= 0.0001 and SOFA: 8.95 ± 3.2 vs. 7.38 ± 3.8, *P *= 0.0001), and higher incidence of shock, mostly more septic shock (47.6% vs. 30.2%, *P *= 0.0007). Follow-up characteristics also differed. Patients who received HES had more frequent secondary shocks (26.8% vs. 11.6%, *P *= 0.0003) with a longer shock duration (5.43 ± 6.1 vs. 2.63 ± 4.2 days, *P *= 0.0001), increased need for vasopressors (80% vs. 48.7%, *P *< 0.0001) and mechanical ventilation (90.5% versus 70.8%, *P *< 0.0001). Duration of mechanical ventilation was also longer (16.9 ± 17.7 vs. 11.9 ± 17.9 days, *P *= 0.008). ICU mortality was 31.1% in the HES group versus 25.8% (*P *= 0.26).

**Table 1:** Cumulative dose of volume expansion after 48 hours, one week and three weeks of ICU stay

Cumulative dose of volume expansion	HES -	HES +	*P*
Crystalloids on day 2 (ml)	3,180 ± 2,171	3,310 ± 2,090	0.56
HES on day 2 (ml)	0	763 ± 595	Inf 0.0001
Albumin on day 2 (ml)	89 ± 374	80 ± 280	0.78
Packed red blood cells on day 2 (n)	0.28 ± 0.86	0.66 ± 1.91	0.019
Crystalloids on day 7	6252 ± 4075	6,587 ± 3,878	0.53
HES on day 7	0	1,031 ± 800	Inf 0.0001
Albumin on day 7	153 ± 457	241 ± 715	0.27
Packed red blood cells on day 7	0.89 ± 1.85	1.4 ± 3.36	0.15
Crystalloids on day 21	10,572 ± 5,930	10,638 ± 6,638	0.96
HES on day 21	0	1,361 ± 1,393	Inf 0.0001
Albumin on day 21	266 ± 743	665 ± 1,587	0.10
Packed red blood cells on day 21	2.77 ± 3.45	3.85 ± 5.53	0.23

HES, hydroxyethylstarch.

**Figure 1:**
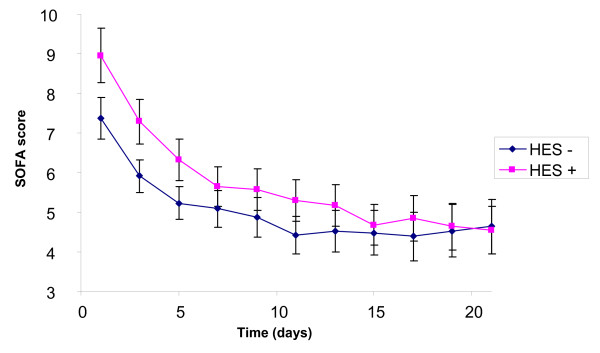
**Evolution of the SOFA score during 21 days with or without HES**. HES, hydroxyethylstarch; SOFA, sepsis related organ failure assessment score.

**Figure 2:**
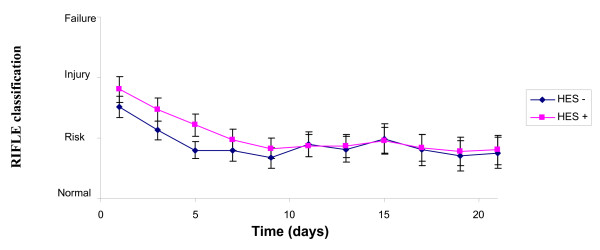
**Evolution of the RIFLE classification during 21 days with or without HES**. HES, hydroxyethylstarch; RIFLE, Risk of renal dysfunction, Injury to the kidney, Failure of kidney function, Loss of kidney function and End-stage kidney disease.

**Figure 3:**
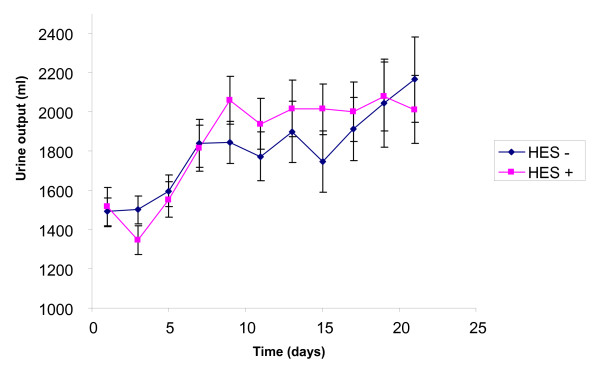
**Evolution of the urine output during 21 days with or without HES**. HES, hydroxyethylstarch.

The SOFA score (Figure 1) was higher in the HES group on admission (8.95 ± 3.2 vs. 7.38 ± 3.8, *P *< 0.0001), decreased during the three-week follow up (*P *< 0.0001) and then the difference disappeared (*P *= 0.23). The RIFLE classification (Figure 2) had the same kinetic: initially higher in the HES group (*P *= 0.007), with initial improvement (*P *< 0.0001), but lack of difference during follow up (*P *= 0.44). The urine output (Figure 3) was similar on admission (1515 ± 1269 ml vs. 1492 ± 987, *P *= 0.37), increased during ICU stay (*P *< 0.0001), without any difference between the groups (*P *= 0.74).

To evaluate the incidence of AKI with regard to HES administration, we separated the patients into two groups: the patients with normal kidney function and those with a RIFLE class 'risk' on admission. The patients who benefited from HES administration had more cardiovascular and respiratory failures on admission, with higher gravity scores (Tables 2 and 3). Presence of AKI risk factors and the development of AKI was similar in patients with or without HES in the two groups. However, patients with HES had a longer duration of shock, mechanical ventilation and ICU stay. There was no difference in ICU mortality (Table 3).

**Table 2:** Baseline characteristics of the patients with normal kidney function and RIFLE class 'risk' on admission broken by HES administration

	Normal kidney function on admission		*P*	RIFLE 'risk' on admission		*P*
	HES - n = 45	HES + n = 28		HES - n = 54	HES + n = 37	
Age (years) (mean ± SD)	59.5 ± 15.9	55.8 ± 17.2	0.4	66.2 ± 13.3	64.1 ± 15.8	0.78
Male sex n(%)	32 (71.1)	20 (71.4)	0.98	41 (75.9)	26 (70.2)	0.55
***Comorbidities***						
Chronic cardiac failure	7 (15.5)	3 (10.7)	0.73	12 (22.2)	5 (13.5)	0.29
More than one cardiovascular event	6 (13.3)	4 (14.3)	1	13 (24)	5 (13.5)	0.21
Diabete mellitus	14 (31.1)	5 (17.9)	0.2	10 (18.5)	10 (27)	0.34
COPD	13 (28.9)	8 (28.6)	0.98	16 (29.6)	12 (32.4)	0.77
Chronic liver failure	7 (15.5)	1 (3.6)	0.14	2 (3.7)	0 (0)	0.51
Chronic alcoholism	8 (17.8)	6 (21.4)	0.7	10 (18.5)	10 (27)	0.34
Chronic renal failure	2 (4.4)	2 (7.1)	0.63	3 (5.5)	3 (8.1)	0.63
Non hematologic malignancy	2 (4.4)	6 (21.4)	0.05	3 (5.5)	2 (5.4)	1
Hematologic malignancy	2 (4.4)	1 (3.6)	1	1 (1.8)	0 (0)	1
Immunosuppression	7 (15.5)	3 (10.7)	0.73	6 (11.1)	2 (5.4)	0.47
***Usual treatments***						
ACE inhibitors/ARB	14 (31.1)	6 (21.4)	0.37	21 (38.9)	15 (40.5)	0.93
Diuretics	15 (33.3)	3 (10.7)	0.05	17 (31.5)	10 (27)	0.6
NSAIDs	2 (4.4)	0 (0)	0.52	3 (5.5)	1 (2.7)	0.64
Hospitalisation before ICU	19 (42.2)	12 (43)	0.96	24 (44.4)	10 (27)	0.09
***Reason for ICU admission***			0.07			0.56
Medicine	41 (91.1)	19 (68)		47 (87)	31 (83.8)	
Planned surgery	1 (2.2)	3 (14.3)		0 (0)	0 (0)	
Urgent surgery	2 (4.4)	4 (10.7)		6 (11.1)	6 (16.2)	
Polytrauma	1 (2.2)	2 (7.1)		1 (1.8)	0 (0)	
***Clinical presentation***						
SAPS II	40.3 ± 15.9	46 ± 19.5	0.32	40.6 ± 14.6	50.3 ± 13.1	0.001
SOFA	6.1 ± 3.6	8.2 ± 3.9	0.03	6.5 ± 3.3	8.4 ± 2.3	0.001
SIRS - sepsis	14 (31.1)	9 (32.1)	0.92	22 (40.7)	11 (29.7)	0.28
Septic shock	8 (17.8)	9 (32.1)	0.16	17 (31.5)	17 (45.9)	0.16
Shock from other etiology	6 (13.3)	5 (17.9)	0.73	5 (9.2)	8 (21.6)	0.1
Bacteremia	4 (8.8)	3 (14.3)	1	6 (11.1)	5 (13.5)	0.75
Platelet count (1000/mm^3^)	245 ± 155	201 ± 159	0.11	291 ± 177	253 ± 164	0.27
pH	7.36 ± 0.1	7.29 ± 0.12	0.02	7.34 ± 0.11	7.3 ± 0.12	0.15
Lactate (meq/l)	1.7 ± 1.1	2.2 ± 1.8	0.43	2.6 ± 1.6	3.4 ± 3.9	0.77
Serum urea (g/l)	0.43 ± 0.3	0.41 ± 0.31	0.68	0.58 ± 0.42	0.63 ± 0.4	0.4
Creatinine (mg/l)	9.9 ± 3.7	10.1 ± 5.9	0.49	12.5 ± 5	13.6 ± 4.8	0.42
PaO_2_/FiO_2_	258 ± 111	202 ± 131	0.04	201 ± 87	193 ± 99	0.6

ACE, angiotensin-converting-enzyme; ARB, angiotensin-II receptor blockers; COPD, chronic obstructive pulmonary disease; FiO2, fraction of inspired oxygen; HES, hydroxyethylstarch; NSAIDs, non-steroidal anti-inflammatory drugs; PaO2, partial pressure of arterial oxygen; RIFLE, Risk of renal dysfunction, Injury to the kidney, Failure of kidney function, Loss of kidney function and End-stage kidney disease; SAPS, simplify acute physiology score; SD, standard deviation; SIRS, systemic inflammatory reaction syndrome; SOFA, sepsis related organ failure assessment score.

**Table 3:** Evolution of the patients with normal kidney function and RIFLE class 'risk' on admission broken by HES administration

	Normal kidney function on admission		*P*	RIFLE "risk" on admission		*P*
	HES - n = 45	HES + n = 28		HES - n = 54	HES + n = 37	
***ICU treatments***						
Catecholamines n (%)	17 (37.7)	19 (67.9)	0.01	27 (50)	27 (73)	0.03
- Norepinephrine	15 (33.3)	18 (64.3)	0.01	26 (48.1)	25 (67.6)	0.07
- Epinephrine	1 (2.2)	3 (10.7)	0.12	2 (3.7)	2 (5.4)	1
- Dobutamine	3 (6.7)	4 (14.3)	0.41	3 (5.5)	4 (10.8)	0.43
Activated protein C	0 (0)	0 (0)	1	0 (0)	0 (0)	1
Hydrocortisone	7 (15.5)	7 (25)	0.7	14 (25.9)	13 (35.1)	0.92
Mechanical ventilation	28 (62.2)	24 (85.7)	0.03	36 (66.7)	33 (89.2)	0.01
Aminoglycosides	8 (17.8)	9 (32.1)	0.16	15 (27.8)	13 (35.1)	0.45
Toxic dose aminoglycosides	1 (2.2)	1 (3.6)	1	0 (0)	0 (0)	1
Glycopeptides	7 (15.5)	6 (21.4)	0.54	13 (24.1)	12 (32.4)	0.38
Toxic dose glycopeptides	1 (2.2)	0 (0)	1	3 (5.5)	1 (2.7)	0.64
Amphotericin B	0 (0)	0 (0)	1	0 (0)	0 (0)	1
Radio-contrast agents	7 (15.5)	4 (14.3)	1	5 (9.2)	5 (13.5)	0.73
Rhabdomyolysis	4 (8.9)	3 (10.7)	1	6 (11.1)	6 (16.2)	0.47
Urinary obstruction	1 (2.2)	0 (0)	1	0 (0)	1 (2.7)	0.4
Chemotherapy	0 (0)	0 (0)	1	0 (0)	1 (2.7)	0.4
***Evolution***						
Shock from day 3 to day 21	3 (6.7)	4 (14.3)	0.41	6 (11.1)	6 (16.2)	0.54
Hemofiltration	0 (0)	2 (7.1)	0.14	3 (5.5)	3 (8.1)	0.68
AKI	5 (11.1)	5 (17.9)	0.49	6 (11.1)	3 (8.1)	0.73
Nosocomial infection	8 (17.8)	8 (28.6)	0.28	11 (20.4)	10 (27)	0.45
Duration of shock (days (mean ± SD))	1.4 ± 2.6	4.2 ± 6.1	0.004	2.1 ± 3.1	3.8 ± 4.2	0.01
Duration of mechanical ventilation (days)	8.4 ± 11.5	16.4 ± 18.6	0.02	13.3 ± 17.2	12.8 ± 16.8	0.1
Duration of hemofiltration (days)	0	1.2 ± 4.3	0.07	0.9 ± 5.2	0.7 ± 2.8	0.38
Duration of ICU stay (days)	12.5 ± 12.7	20.2 ± 20.3	0.09	14.8 ± 16.6	15.4 ± 16.6	0.27
ICU death	9 (20)	7 (25)	0.62	12 (22.2)	12 (32.4)	0.28

AKI, acute kidney injury; HES, hydroxyethylstarch; RIFLE, Risk of renal dysfunction, Injury to the kidney, Failure of kidney function, Loss of kidney function and End-stage kidney disease; SD, standard deviation.

## Discussion

Considering all the patients admitted to the ICU for more than 72 hours, HES volume expansion did not worsen the RIFLE class or SOFA score despite higher RIFLE and SOFA on admission in patients receiving HES. The groups of patients with no kidney dysfunction or at risk of kidney injury on admission who received HES were more severely ill; in particular, they had more cardiovascular and respiratory failures. Development of AKI was not different.

Published AKI incidence in ICU ranges from 5 to 25% with a 40 to 80% mortality rate [19,24-27]. Differences in the literature come from the variability of AKI definition [28]. This heterogeneity led to the creation of an AKI classification, called RIFLE, by an international consensus [23]. This classification has been validated in retrospective studies, which also correlated the RIFLE to mortality [29-31].

No study has demonstrated the superiority of colloids on crystalloids on morbimortality [3] and renal toxicity of HES has been controversial for a long time. The first studies suggesting a toxicity were performed in renal transplantation. In a 1993 retrospective study, Legendre and colleagues discovered 80% osmotic lesions in renal transplant recipients who received HES (200 kDa/0.62) versus 14% before their utilisation [3]. Then, Cittanova and colleagues [4] prospectively compared HES (200 kDa/0.62) with gelatins as volume expander in the resuscitation of donor patients with brain death. RRT need on day 8 after renal transplantation was 33% in the HES group compared with 5% in the gelatins group. However, these studies were criticized for their methodology and unconfirmed by further studies [11,12].

Two double-blind multicenter randomized studies found a nephrotoxicity of HES in ICU patients [5,6]. In a study of 129 patients with severe sepsis and septic shock [5], Schortgen and colleagues demonstrated that HES 6% (200 kDa/0.6) was an independent risk factor for AKI (odds ratio (OR) = 2.57) compared with gelatins. More recently, the VISEP study [6] compared volume expansion with ringer's lactate versus HES (200 kDa/0.5) for severe sepsis and septic shock. The use of HES (200 kDa/0.5) was associated with an increased incidence of renal failure (34.9% versus 22.8%, *P *= 0.002) and RRT need (31% vs. 18.8%, *P *= 0.001), and with a dose-effect relation.

Schortgen and colleagues, in a prospective cohort study of 1013 septic shock patients, showed a 2.48 renal failure risk increase with hyperoncotic colloid compared with crystalloids [7]. This study used 'modern' HES solutions with a low molecular weight and low substitution rate (HES 6% (130 kDa/0.4), identical to our study. Once again, a dose-effect relation was found and renal failure occurred when patients received more than 2000 ml of HES during 36 hours. The study also found an increased ICU and day-28 mortality associated with HES, and an AKI increase with 20% albumin. Another recent study in cardiac surgery found that HES 250 kDa/0.45 was an independent AKI risk factor with a dose-dependant relation (OR = 1.08/ml/kg infused) [10].

A recent review performed by the Cochrane group compiled 34 randomized clinical trials (2607 patients) comparing HES with other fluid therapies and found a 1.5 fold risk of developing AKI with HES, with an increased risk in septic patients [32]. However, the results are limited by the different definitions of AKI and the authors insisted on the use of validated AKI criteria.

We wanted to evaluate the impact of HES volume expansion on renal function in our unit. Vascular expansion with HES was not applied according to a protocol, but, looking at our results, it seems mostly used for more critically ill patients: patients with HES had higher severity scores and more frequent mechanical ventilation and vasopressors. Whereas the RIFLE class was also higher in the patients who received HES, the evolution of renal function did not demonstrate any deleterious effect of HES. Moreover, some could argue that HES administered in small volumes is in fact protective, but our study did not have the power to draw this conclusion and only a well-designed prospective randomized study could prove a beneficial effect of small-volume HES administration.

Similar results have already been published. A large prospective multicenter study of more than 3000 ICU patients found that HES administration did not increase the need for RRT [13]. An important difference between this study and the VISEP study was the HES volume administered. In the VISEP study, the mean cumulative dose of HES 200 kDa/0.5 was 70.4 ml/kg and volume expansion was performed exclusively with ringer's lactate or HES. This is far above the manufacturer's recommendations of 33 ml/kg/day on day 1, then 20 ml/kg/day. In the study by Sakr and colleagues [13], the cumulative dose was much lower, around 1000 ml in the first 48 hours, similar to the volume we used. In the post-operative setting, other studies did not find deleterious effects of HES, even in patients with chronic renal dysfunction [16,17]. Once again, small volumes of HES were administered.

The HES used in prospective randomized studies were intermediate molecular weight. In our unit, the HES is of small molecular weight (130 kDa/0.4) with low substitution rate. Some studies suggest these 'modern' HES are less nephrotoxic, in renal transplantation [33], and in neurological intensive care [14]. The latest study, in patients with cranial trauma, did not find any difference on renal function between patients receiving as much as 70 ml/kg/day HES 130 kDa/0.4 versus HES 200 kDa/0.5 33 ml/kg/day with additional albumin. A recent review [34] confirmed the deleterious effects of older HES preparations with high molecular weight and a high degree of substitution administered in high volume and the interest in tetrastarch solutions, probably less (or not at all) nephrotoxic. According to these studies, the main mechanism of HES-induced kidney injury is probably hyperoncoticity: kidney lesions called osmotic nephrosis associated with a decrease of glomerular filtration pressure secondary to a more rapid increase in intracapillary oncotic pressure than hydrostatic pressure. This theory well explains the nephrotoxicity associated with HES and serum albumin in a recent multicenter observational study [7]. This might also explain the low toxicity of 'modern' HES solutions.

### Study limitations

We performed a retrospective practice survey. Our results do not support that HES are not nephrotoxic, but suggest that in our daily practice, their use is not directly associated with AKI. However, our results must be taken with caution because this survey has numerous biases. First, our groups are not homogeneous: HES group is composed of more severe patients. As HES administration was not conducted according to a protocol, attending physicians could have generated a systematic bias with no HES use when patients had renal failure or were at risk of AKI. This seems improbable, because patients who received HES had initial higher RIFLE classes and SOFA scores. Second, considering the small HES volumes administered, our study could lack power to demonstrate a deleterious effect of HES on renal function. Third, some could argue that we assessed AKI based on the RIFLE classification, commonly used at the time we designed our study, instead of the more recent Acute Kidney Injury Network (AKIN) staging system [35]. This has probably no influence on our results, as RIFLE 'injury' and 'failure' classes correspond to AKIN staging system 2 and 3. Finally, as we used low substitution rate HES, our results cannot be extrapolated to other types of HES.

## Conclusions

The nephrotoxicity of high molecular weight and high substitution rate HES administered at high posology in patients with severe sepsis and septic shock is demonstrated by multiple studies. However, we suggest that volume expansion with 'modern' HES and much lower volumes than those recommended, is not associated with AKI, even in patients with kidney dysfunction on admission.

## Key messages

• Resuscitation with low volume of HES 130 kDa/0.4 is not associated with AKI.

## Abbreviations

ACE: angiotensin-converting-enzyme; AKI: acute kidney injury; ARB: angiotensin-II receptor blockers; HES: hydroxyethylstarch; NSAIDs: non-steroidal anti-inflammatory drugs; OD: odds ratio; RIFLE: Risk of renal dysfunction, Injury to the kidney, Failure of kidney function, Loss of kidney function and End-stage kidney disease; RRT: renal replacement therapy; SAPS: simplify acute physiology score; SOFA: sepsis related organ failure assessment score.

## Competing interests

The authors declare that they have no competing interests.

## Authors' contributions

NB and RD collected the data. NB, RD, JL, SA, AM, AC, HG and OL drafted the manuscript. PD performed the statistical analysis. All the authors read and approved this manuscript.
